# Dyadic associations between perceived family resilience and fear of disease progression among reproductive-age patients with cervical cancer and their spouses: a cross-sectional study

**DOI:** 10.3389/fonc.2026.1927068

**Published:** 2026-10-28

**Authors:** Mei-Gui Peng, Zi-Yi Yuan, Long-Xian Liu, Shuang Lu, Mei-Xia Ye

**Affiliations:** Shenzhen Hospital (Futian) of Guangzhou University of Chinese Medicine, Sixth Clinical Medical College of Guangzhou University of Chinese Medicine, Shenzhen, China

**Keywords:** actor–partner interdependence model, cervical cancer, fear of disease progression, perceived family resilience, reproductive age, spouse

## Abstract

**Background:**

Fear of disease progression (FoP) and perceived family resilience may be interrelated within patient–spouse dyads, but member-specific and cross-partner associations in cervical cancer are insufficiently characterized.

**Methods:**

This cross-sectional dyadic study included 208 reproductive-age patients with cervical cancer and their spouses. FoP was assessed using the Fear of Progression Questionnaire–Short Form (FoP-Q-SF) in patients and its partner version (FoP-Q-SF/P) in spouses; perceived family resilience was assessed using the Chinese version of the Family Resilience Questionnaire (FaRE). Paired comparisons, intraclass correlation analysis, Pearson correlations, and actor–partner interdependence model (APIM) analyses were conducted for distinguishable patient–spouse dyads. APIMs were estimated without and with adjustment for patient and spouse age and education, clinical stage, metastasis/recurrence status, and childbirth history.

**Results:**

Patients reported higher FoP scores than spouses (46.88 ± 9.69 vs. 31.84 ± 4.79; t(207) = 20.71, P < 0.001). A total of 190 patients (91.3%) and 124 spouses (59.6%) scored ≥34; this patient-derived threshold was applied descriptively to spouses. Patient-perceived family resilience was lower than spouse-perceived family resilience (83.13 ± 16.20 vs. 88.22 ± 15.13; t(207) = −3.95, P < 0.001), with an absolute-agreement intraclass correlation coefficient (ICC) of 0.283 (95% confidence interval [CI] 0.156–0.399). In the adjusted APIM, patient-perceived family resilience was negatively associated with patient FoP (β = −0.179, P = 0.001) and spouse FoP (β = −0.544, P < 0.001). Spouse-perceived family resilience was negatively associated with patient FoP (β = −0.176, P = 0.003), whereas its association with spouse FoP was not statistically significant (β = −0.126, P = 0.053).

**Conclusions:**

In the adjusted APIM, higher patient-perceived family resilience was associated with lower FoP in both patients and spouses, and higher spouse-perceived family resilience was associated with lower patient FoP. Further research should evaluate approaches to assessing and managing FoP that include both patients and spouses.

## Introduction

Cervical cancer remains a major global public health concern, with a disproportionate burden in low- and middle-income countries (1). Although advances in human papillomavirus (HPV) vaccination and cervical screening have improved prevention, cervical cancer continues to affect a substantial number of women during their reproductive years. The burden among younger women is particularly relevant because diagnosis during this life stage may coincide with ongoing fertility and family-planning goals (2).

For women of reproductive age, cervical cancer represents not only a serious health condition but also a potential disruption to fertility, reproductive function, and future family planning. Fertility and parenthood remain important concerns for many young women with gynecologic cancers, and impaired fertility may adversely affect psychological well-being and quality of life (3). Cancer-directed treatments, including surgery, pelvic radiotherapy, and chemotherapy, may compromise reproductive potential, making reproductive risk assessment and fertility counseling important components of care for patients of reproductive age (4).

Fear of disease progression (FoP) refers to concerns about the possibility that cancer may recur, progress, or otherwise worsen. FoP is a substantial psychological concern among patients with cancer and has been associated with anxiety, distress, and poorer quality of life (5). These concerns are not confined to patients. Spouses may also experience FoP while coping with uncertainty about the patient’s disease and future health. Recent dyadic research in cancer populations has shown that FoP and related psychological responses may be associated across members of the same patient–partner dyad, supporting assessment at the couple rather than solely the individual level (6).

Family resilience reflects the adaptive processes through which families respond to significant adversity by drawing on shared beliefs, communication, coping, and available resources (7). Recent evidence in adult oncology indicates that perceived family resilience is associated with psychosocial outcomes in both patients and family members and that patients and family members may provide related but distinct evaluations of their family’s resilience (8). Nevertheless, research on family resilience in cancer has largely examined patient or caregiver outcomes separately, and dyadic evidence remains comparatively limited (9). In particular, little is known about whether patient- and spouse-perceived family resilience are associated with their own and their partner’s FoP among couples facing cervical cancer during reproductive age. The actor–partner interdependence model (APIM) allows actor and partner associations to be estimated simultaneously while accounting for the non-independence of observations within distinguishable dyads (10). Therefore, this study used APIM to examine the individual and cross-partner associations between perceived family resilience and FoP among cervical cancer patients of reproductive age and their spouses. The findings may inform couple-centered psychological assessment and supportive care in this population.

## Methods

### Study design and participants

This cross-sectional dyadic study was conducted at Shenzhen Hospital (Futian) of Guangzhou University of Chinese Medicine, China, from February 7 to June 30, 2026. Cervical cancer patients of reproductive age and their spouses were recruited from the hospital’s inpatient wards, outpatient clinics, and affiliated community health centers using convenience sampling.

Patients were eligible if they: (1) had histopathologically confirmed cervical cancer; (2) were aged 18–49 years; (3) were aware of their diagnosis and clinically stable; (4) were able to communicate effectively and provide independent responses to the questionnaires; and (5) provided written informed consent. Spouses were eligible if they: (1) were aware of the patient’s diagnosis; (2) were identified as the primary caregiver and provided at least 4 h of direct hands-on care to the patient per day; and (3) agreed to participate. Patients and spouses were excluded if they had a severe psychiatric disorder or cognitive impairment or were concurrently participating in another clinical study. Patients with another major malignancy were also excluded.

The study was conducted in accordance with the Declaration of Helsinki and was approved by the Ethics Committee of Shenzhen Hospital (Futian) of Guangzhou University of Chinese Medicine (Approval No. GYL(KY)-2026-027). All participants provided written informed consent before participation.

### Sample size

No formal *a priori* power analysis was performed to determine the sample size required to detect expected actor and partner associations. Sample-size considerations focused on the parameterization of the unadjusted observed-variable APIM, which included 10 freely estimated covariance-structure parameters: four actor and partner regression paths, two variances of the exogenous family resilience variables, one covariance between these variables, two residual variances of FoP, and one residual covariance between patient and spouse FoP. The final analytic sample comprised 208 patient–spouse dyads, with dyads treated as the independent sampling units. The parameter count refers to the unadjusted model; the covariate-adjusted model included additional parameters. These considerations describe model parameterization and estimability but do not establish statistical power or guarantee estimation stability (11).

### Measures

#### Sociodemographic and clinical characteristics

A structured questionnaire developed by the research team recorded patient age, residence, education, employment status, clinical stage, pathological type, metastasis/recurrence status, medical insurance, childbirth history, and religious belief. Spouse variables included age, education, employment status, and daily caregiving time. Clinical stage was obtained from the medical record based on the International Federation of Gynecology and Obstetrics (FIGO) staging system and grouped as stages I–II and III–IV for analysis.

#### Fear of disease progression in patients

FoP was assessed with the 12-item Fear of Progression Questionnaire-Short Form (FoP-Q-SF), with each item rated from 1 to 5 and total scores ranging from 12 to 60; higher scores indicate greater FoP. The original short form and Chinese validation support its use in cancer populations (12, 13). A prespecified total score of ≥34 was used as a patient-derived reference threshold for elevated FoP, based on previous adult cancer research (14). Cronbach’s α in the present patient sample was 0.928.

#### Fear of disease progression in spouses

Spouses completed the 12-item partner version (FoP-Q-SF/P), which uses the same response range and total-score range but frames items around concern about the ill partner (15). Cronbach’s α in the present spouse sample was 0.849. For descriptive reporting, the same patient-derived reference threshold of ≥34 was applied to spouses. However, this threshold has not been independently validated for the FoP-Q-SF/P among spouses of reproductive-age patients with cervical cancer. The percentage of spouses scoring ≥34 is therefore reported as descriptive threshold exceedance rather than as the prevalence of clinically relevant FoP.

#### Perceived family resilience

Family resilience was measured using the 24-item Chinese version of the Family Resilience Questionnaire (FaRE), which includes communication and cohesion, perceived social support, family coping, and religious belief. Items are rated on a 7-point scale and total scores range from 24 to 168, with higher scores indicating greater perceived family resilience (16). Cronbach’s α was 0.838 for patient reports and 0.900 for spouse reports. Patient and spouse FaRE totals were analyzed separately as member-specific perceptions of the shared family context.

### Data collection

After institutional permission was obtained, all investigators received standardized training in participant screening and questionnaire administration. Eligible patient–spouse dyads were screened according to the predefined inclusion and exclusion criteria and were informed of the study purpose, procedures, confidentiality protections, voluntary nature of participation, and right to withdraw. After written informed consent was obtained, patients and spouses completed the questionnaires independently in private rooms and separately from one another to minimize mutual influence on their responses. An investigator was available to clarify questionnaire instructions when necessary without suggesting or interpreting responses. For participants with reading difficulties, trained investigators read the items aloud in a neutral manner and recorded the participants’ responses verbatim. Completed questionnaires were reviewed immediately for completeness, and participants were invited to complete inadvertently omitted items when appropriate. Questionnaires were distributed to 210 eligible patient–spouse dyads. Two dyads withdrew during questionnaire completion and were excluded from the analysis. The remaining 208 dyads provided valid questionnaires and were included in the analysis, yielding a valid questionnaire rate of 99.05%.

### Statistical analysis

Analyses were conducted using IBM SPSS Statistics version 27.0 and IBM SPSS Amos version 24.0. The final analytic dataset contained no missing values for any variables included in the analyses, and no data imputation was performed. Continuous variables were summarized as mean ± standard deviation (SD), and categorical variables as frequencies and percentages. Internal consistency was assessed using Cronbach’s α. Differences in FoP and perceived family resilience between patients and spouses were examined using paired-samples t tests and reported with mean paired differences and 95% confidence intervals (CIs). The proportions of patients and spouses with FoP scores ≥34 were calculated descriptively; for spouses, this was an exploratory application of the patient-derived reference threshold. Correlation and APIM analyses used continuous FoP scores. Agreement between patient- and spouse-reported family resilience was assessed using a two-way mixed-effects, absolute-agreement, single-measure intraclass correlation coefficient [ICC(A,1)] with a 95% CI obtained from 20,000 dyad-level bootstrap resamples. Pearson correlation coefficients were calculated for the four focal variables.

The APIM was estimated in Amos using maximum likelihood within an observed-variable structural equation modeling framework for distinguishable patient–spouse dyads. Actor associations represented associations between each member’s perceived family resilience and their own FoP, whereas partner associations represented associations between one member’s perceived family resilience and the other member’s FoP. Patient- and spouse-perceived family resilience were entered simultaneously as exogenous predictors of both FoP outcomes. The two family-resilience variables were allowed to covary, and the residuals of patient and spouse FoP were allowed to covary.

Unadjusted and covariate-adjusted APIMs were estimated. The covariate-adjusted model included patient age, spouse age, patient and spouse education, clinical stage, metastasis/recurrence status, and childbirth history as predictors of both FoP outcomes. Education was treated as a categorical variable using indicator coding. Exogenous predictors in the adjusted model were allowed to covary. Path estimates were reported as unstandardized coefficients (B), standard errors (SEs), 95% CIs, standardized coefficients (β), and P values, together with R² for patient and spouse FoP. The observed-variable APIM was just-identified (df = 0); therefore, global model-fit indices were not interpreted. All tests were two-tailed, with P < 0.05 considered statistically significant.

## Results

### Participant characteristics

The analysis included 208 patient-spouse dyads. The mean age was 42.49 ± 5.04 years for patients and 45.48 ± 8.34 years for spouses. Sociodemographic and clinical characteristics are presented in **Table 1**.

**Table 1:** Sociodemographic and clinical characteristics of the 208 patient–spouse dyads.

Characteristic	Patient, n (%) or mean ± SD	Spouse, n (%) or mean ± SD
Age, years	42.49 ± 5.04	45.48 ± 8.34
Residence		
Urban	181 (87.0)	–
Rural	27 (13.0)	–
Education		
Junior high school or below	56 (26.9)	33 (15.9)
High school/junior college	102 (49.0)	136 (65.4)
Bachelor’s degree or above	50 (24.0)	39 (18.8)
Employment status		
Employed	145 (69.7)	193 (92.8)
Not employed	63 (30.3)	15 (7.2)
Clinical stage		
I-II	103 (49.5)	–
III-IV	105 (50.5)	–
Pathological type		
Squamous cell carcinoma	92 (44.2)	–
Adenocarcinoma	96 (46.2)	–
Other	20 (9.6)	–
Metastasis/recurrence		
Yes	123 (59.1)	–
No	85 (40.9)	–
Medical insurance		
Urban employee insurance	136 (65.4)	–
Urban/rural resident insurance	34 (16.3)	–
New rural cooperative medical scheme	7 (3.4)	–
Self-pay	2 (1.0)	–
Other	29 (13.9)	–
Childbirth history		
Previous childbirth	167 (80.3)	–
No previous childbirth	41 (19.7)	–
Religious belief		
Yes	17 (8.2)	–
No	191 (91.8)	–
Daily caregiving time		
4 to <8 h	–	169 (81.3)
8 to <12 h	–	35 (16.8)
12–24 h	–	4 (1.9)

Dashes indicate information not reported. Percentages may not sum to 100% because of rounding. SD, standard deviation.

### FoP and perceived family resilience

Patients reported higher FoP scores and lower perceived family resilience scores than their spouses (**Table 2**). A total of 190 patients (91.3%) and 124 spouses (59.6%) had FoP scores ≥34. The spouse percentage represents descriptive exceedance of a patient-derived reference threshold. The absolute-agreement ICC between patient- and spouse-perceived family resilience scores was 0.283 (95% CI 0.156–0.399).

**Table 2:** Comparison of FoP and perceived family resilience between patients and spouses.

Variable	Patients, mean ± SD	Spouses, mean ± SD	Mean paired difference	95% CI	t (df)	P
FoP	46.88 ± 9.69	31.84 ± 4.79	15.04	13.61 to 16.47	20.71 (207)	<0.001
Perceived family resilience	83.13 ± 16.20	88.22 ± 15.13	−5.09	−7.63 to −2.55	−3.95 (207)	<0.001

Mean paired differences were calculated as patient minus spouse scores. CI, confidence interval; FoP, fear of disease progression; SD, standard deviation. Paired-samples t tests were used.

### Correlations among focal variables

Patient- and spouse-perceived family resilience were each negatively correlated with FoP in both dyad members (all P < 0.001). Patient- and spouse-perceived family resilience were positively correlated (r = 0.298, P < 0.001), whereas patient and spouse FoP were not significantly correlated (r = 0.078, P = 0.265). The full correlation matrix is presented in **Table 3**.

**Table 3:** Pearson correlations among perceived family resilience and FoP in patient–spouse dyads.

Variable	Mean ± SD	1	2	3	4
1. Patient-perceived family resilience	83.13 ± 16.20	1			
2. Patient FoP	46.88 ± 9.69	−0.329***	1		
3. Spouse-perceived family resilience	88.22 ± 15.13	0.298***	−0.309***	1	
4. Spouse FoP	31.84 ± 4.79	−0.579***	0.078	−0.325***	1

Values are Pearson correlation coefficients (r). FoP, fear of disease progression. ***P < 0.001.

### Actor–partner interdependence models

In the unadjusted APIM, all actor and partner associations were negative and statistically significant (**Table 4**). After covariate adjustment, patient-perceived family resilience was negatively associated with patient FoP (β = −0.179, P = 0.001) and spouse FoP (β = −0.544, P < 0.001). Spouse-perceived family resilience was negatively associated with patient FoP (β = −0.176, P = 0.003), whereas its association with spouse FoP was not statistically significant (β = −0.126, P = 0.053). The full covariate-adjusted model accounted for 51.6% of the variance in patient FoP and 38.8% of the variance in spouse FoP. Full estimates for both models are presented in **Table 4**, and the covariate-adjusted APIM is illustrated in **Figure 1**. **Figure 2** summarizes the four actor and partner associations in both models using unstandardized coefficients and their 95% CIs.

**Table 4:** Unadjusted and covariate-adjusted APIM estimates for associations between perceived family resilience and FoP.

Model	Path	B	SE	95% CI	β	P	Outcome R²
Unadjusted	Patient FR → Patient FoP	-0.156	0.040	-0.235 to -0.076	-0.260	<0.001	0.157
	Spouse FR → Patient FoP	-0.148	0.043	-0.233 to -0.063	-0.232	<0.001	
	Spouse FR → Spouse FoP	-0.053	0.019	-0.090 to -0.016	-0.167	0.005	0.361
	Patient FR → Spouse FoP	-0.157	0.017	-0.191 to -0.122	-0.529	<0.001	
Covariate-adjusted	Patient FR → Patient FoP	-0.107	0.032	-0.171 to -0.043	-0.179	0.001	0.516
	Spouse FR → Patient FoP	-0.113	0.037	-0.185 to -0.040	-0.176	0.003	
	Spouse FR → Spouse FoP	-0.040	0.020	-0.080 to 0.001	-0.126	0.053	0.388
	Patient FR → Spouse FoP	-0.161	0.018	-0.196 to -0.125	-0.544	<0.001	

APIM, actor–partner interdependence model; B, unstandardized coefficient; SE, standard error; CI, confidence interval; β, standardized coefficient; FoP, fear of disease progression; FR, perceived family resilience. The covariate-adjusted model included patient age, spouse age, patient education, spouse education, clinical stage, metastasis/recurrence status, and childbirth history. Outcome R² values refer to the full model.

**Figure 1:**
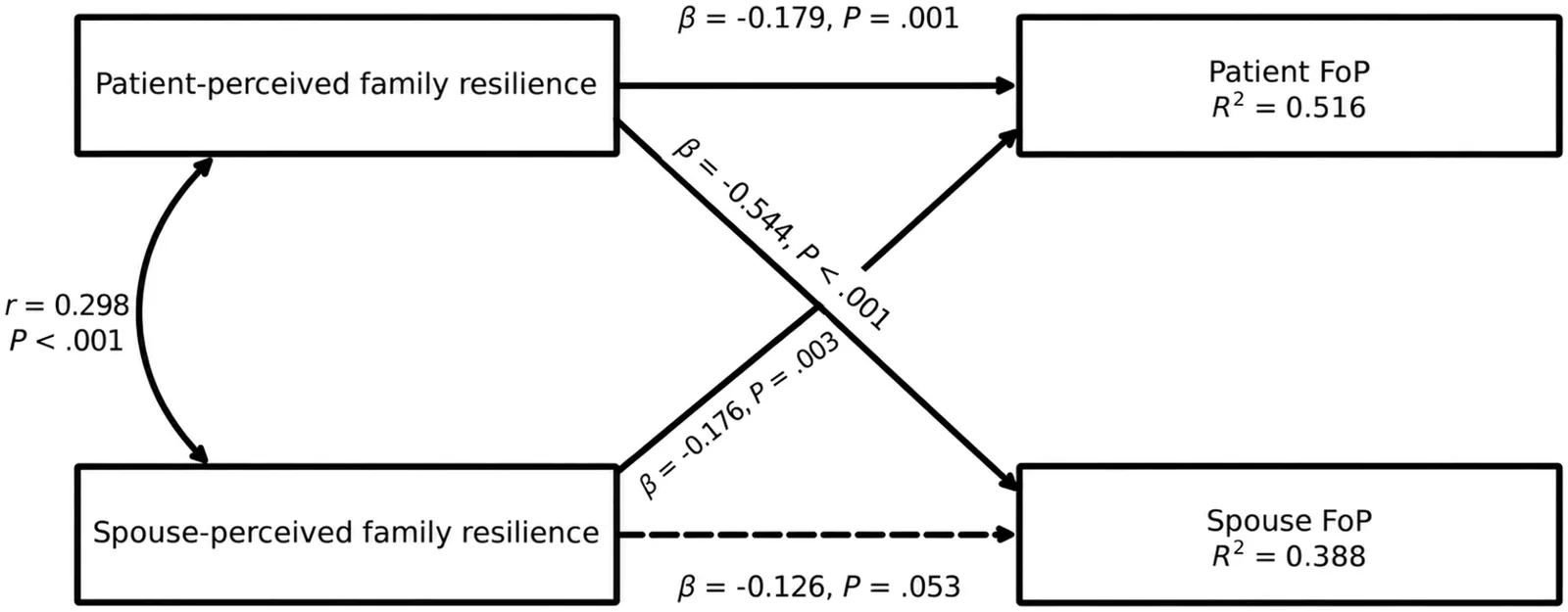
Covariate-adjusted actor–partner interdependence model of perceived family resilience and fear of disease progression. Standardized path coefficients (β) are shown. Solid arrows indicate statistically significant associations (P < 0.05), whereas the dashed arrow indicates a nonsignificant association. R² values refer to the full covariate-adjusted model. Arrows represent specified statistical associations and do not establish temporal or causal relationships. Covariate paths and the correlated residuals of patient and spouse FoP are omitted for clarity.

**Figure 2:**
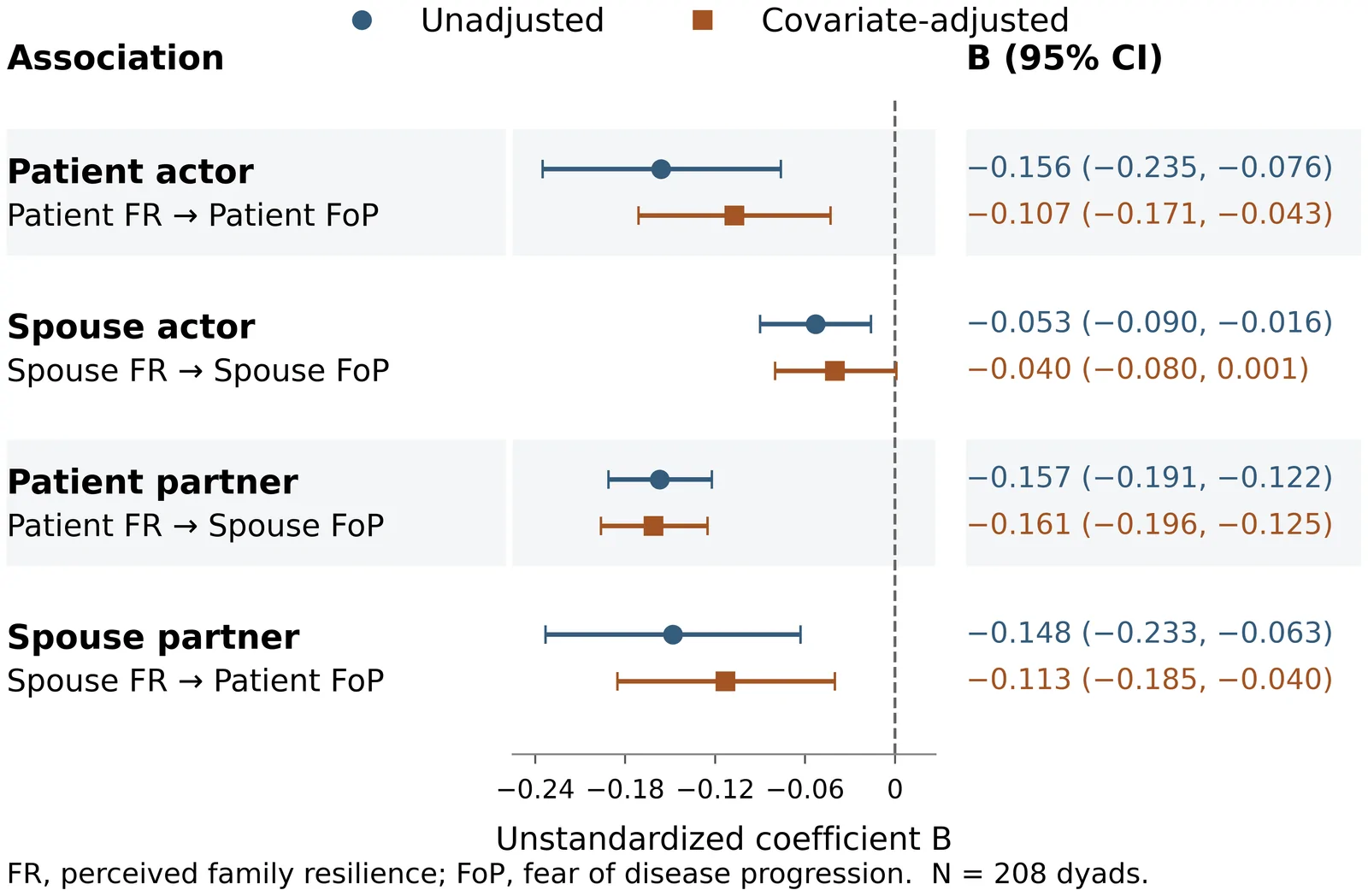
Actor and partner associations between perceived family resilience and FoP in the unadjusted and covariate-adjusted APIMs. Points indicate unstandardized coefficients B, expressed as estimated differences in FoP score associated with a one-point higher perceived family resilience score; horizontal bars indicate 95% CIs. Circles represent the unadjusted model and squares the covariate-adjusted model. The vertical dashed line denotes B = 0. Estimates are reproduced from **Table 4** (N = 208 dyads). The adjusted model included patient age, spouse age, patient education, spouse education, clinical stage, metastasis/recurrence status, and childbirth history. FR, perceived family resilience; FoP, fear of disease progression.

## Discussion

This study examined the associations between perceived family resilience and FoP from both patient and spouse perspectives in cervical cancer dyads. Patients reported higher FoP scores but lower perceived family resilience scores than their spouses. Patient and spouse perceptions of family resilience showed relatively low absolute agreement. In the unadjusted APIM, all actor and partner associations were negative. After adjustment for the measured sociodemographic and clinical covariates, patient-perceived family resilience remained negatively associated with both patient and spouse FoP, and spouse-perceived family resilience remained negatively associated with patient FoP. The spouse actor association was no longer statistically significant after adjustment.

Using the patient-derived ≥34 reference threshold, 91.3% of patients and 59.6% of spouses scored at or above this value. Although fear of cancer recurrence (FCR) is conceptually related to but not identical to FoP, a systematic review and meta-analysis of caregiver FCR found that fear related to recurrence or progression is also common among caregivers, with approximately 48% meeting thresholds used for clinically significant fear in previous studies; however, the authors also noted that clinically established cutoffs for caregiver populations remain limited (17). Differences in instruments, cutoff definitions, and study populations preclude a direct comparison of that pooled estimate with the spouse percentage reported here. Previous research across breast, prostate, and laryngeal cancer couples has also demonstrated sex- and role-specific differences in recurrence-related fear, indicating that the relative levels reported by patients and partners are not uniform across cancer contexts (18). In the present sample, the correlation between patient and spouse FoP scores was small and not statistically significant (r = 0.078, P = 0.265). A study of Thai gynecologic-cancer survivor–caregiver dyads reported a moderate positive correlation (r = 0.37) (19). Differences in populations and measurement should be considered when interpreting these findings.

Spouses reported higher perceived family resilience than patients, while the absolute agreement between their scores was relatively low. A recent study of colorectal cancer couples similarly found higher family resilience scores among spousal caregivers and demonstrated that patient and spouse assessments contained both shared and discrepant information (20). Recent dyadic research in patients with gynecological cancer and their spouses has likewise modeled family resilience separately for the two dyad members within an APIM framework, supporting the relevance of retaining member-specific information in couple-level analyses (21). In the present study, the ICC of 0.283 suggested limited absolute agreement; patient and spouse reports should therefore not be assumed to be interchangeable.

The negative associations between perceived family resilience and FoP are broadly consistent with previous oncology evidence. In a cross-sectional study of 392 breast cancer survivors and their spouses, family resilience was negatively associated with FCR in both survivors and spouses, although that study assessed family resilience primarily from the survivor perspective and used a different structural model (22). He et al. recently reported a negative association between family resilience and psychological distress in 358 patients with gynecological cancer (23). That study assessed patient reports and general psychological distress; the present findings complement this patient-only evidence by examining FoP in both patients and spouses and incorporating their separate reports of perceived family resilience within an APIM. Earlier dyadic research in cancer couples has also shown that family resilience can exhibit both actor and partner associations with psychosocial outcomes, indicating that member-specific perceptions may contain information relevant to outcomes reported by both members of the couple (24). A recent APIM study of cervical cancer couples also reported actor and partner associations between dyadic coping and psychological resilience (25).

In the covariate-adjusted model, the patient actor association and both partner associations were statistically significant, whereas the spouse actor association was not. The adjusted spouse actor estimate was negative, but its 95% CI included zero (B = −0.040, 95% CI −0.080 to 0.001; β = −0.126, P = 0.053). This interval should be considered when interpreting the nonsignificant result, which does not establish the absence of a spouse actor association. Differences in statistical significance do not establish differences between path coefficients. The reported analyses do not establish differences in association strength between patients and spouses.

A recent qualitative study by Zhang et al. identified both shared and individual uncertainties about recurrence, treatment, and future planning among gynecological cancer couples (26). Although that exploratory study included only four older couples and predominantly ovarian cancer, its findings highlight differences in patients’ and partners’ psychosocial experiences. These observations provide further context for assessing both members separately.

These findings are relevant to dyadic psychosocial assessment. Assessing only the patient may overlook spouses’ FoP concerns, while relying on one family member’s family-resilience score may not adequately represent the other member’s perception. Couple-oriented approaches have shown promise in related cancer populations: a randomized controlled trial among young Chinese breast cancer patients and their spouses found that couple skills training reduced patient FCR and improved cancer-related communication over follow-up (27). The present associations provide a rationale for including both members in psychosocial assessment and for testing couple-oriented approaches in cervical cancer care. They do not establish that increasing perceived family resilience would itself cause reductions in FoP.

### Strengths and limitations

This study incorporated reports from both patients and spouses and used a distinguishable-dyad APIM to examine member-specific and cross-partner associations while accounting for dyadic non-independence. Several limitations should be considered. First, the cross-sectional design does not establish temporal ordering or causal relationships between perceived family resilience and FoP. Second, convenience sampling within a single hospital network, including its inpatient wards, outpatient clinics, and affiliated community health centers, may limit generalizability to other clinical settings. Spouses were primary caregivers providing at least 4 h of care per day, which may also limit applicability to spouses with different caregiving roles. Third, all focal variables were assessed by self-report during the same assessment period, which may have introduced common-method bias and response-related measurement error. Patients and spouses completed differently worded FoP instruments, so between-member score comparisons also warrant caution. Fourth, the ≥34 reference threshold has not been independently validated for the FoP-Q-SF/P in this spouse population, so the proportion of spouses above the threshold should not be interpreted as the prevalence of clinically relevant FoP. Fifth, no formal *a priori* power analysis was performed for the actor and partner associations; therefore, power to detect small associations was not established. The nonsignificant adjusted spouse actor estimate should be interpreted with its 95% CI rather than as evidence that the association is absent. Finally, although the covariate-adjusted model accounted for the measured sociodemographic and clinical characteristics included in the study, treatment characteristics were not available for adjustment, and residual confounding cannot be excluded.

## Conclusion

In the covariate-adjusted APIM, higher patient-perceived family resilience was associated with lower FoP scores in both patients and spouses. Higher spouse-perceived family resilience was associated with lower patient FoP scores, while its association with spouse FoP did not reach statistical significance. Patient and spouse perceptions of family resilience showed limited absolute agreement. Future longitudinal and intervention studies should clarify temporal relationships and evaluate whether approaches that include both members can improve psychological outcomes.

## Data Availability

The raw data supporting the conclusions of this article will be made available by the authors, without undue reservation.
